# Supplementary figures and images for: Ectopic Overexpression of Pineapple Transcription Factor AcWRKY31 Reduces Drought and Salt Tolerance in Rice and Arabidopsis
Source: Int J Mol Sci. 2022 Jun 3;23(11):6269. doi: 10.3390/ijms23116269 (PMC9181287; doi:10.3390/ijms23116269)

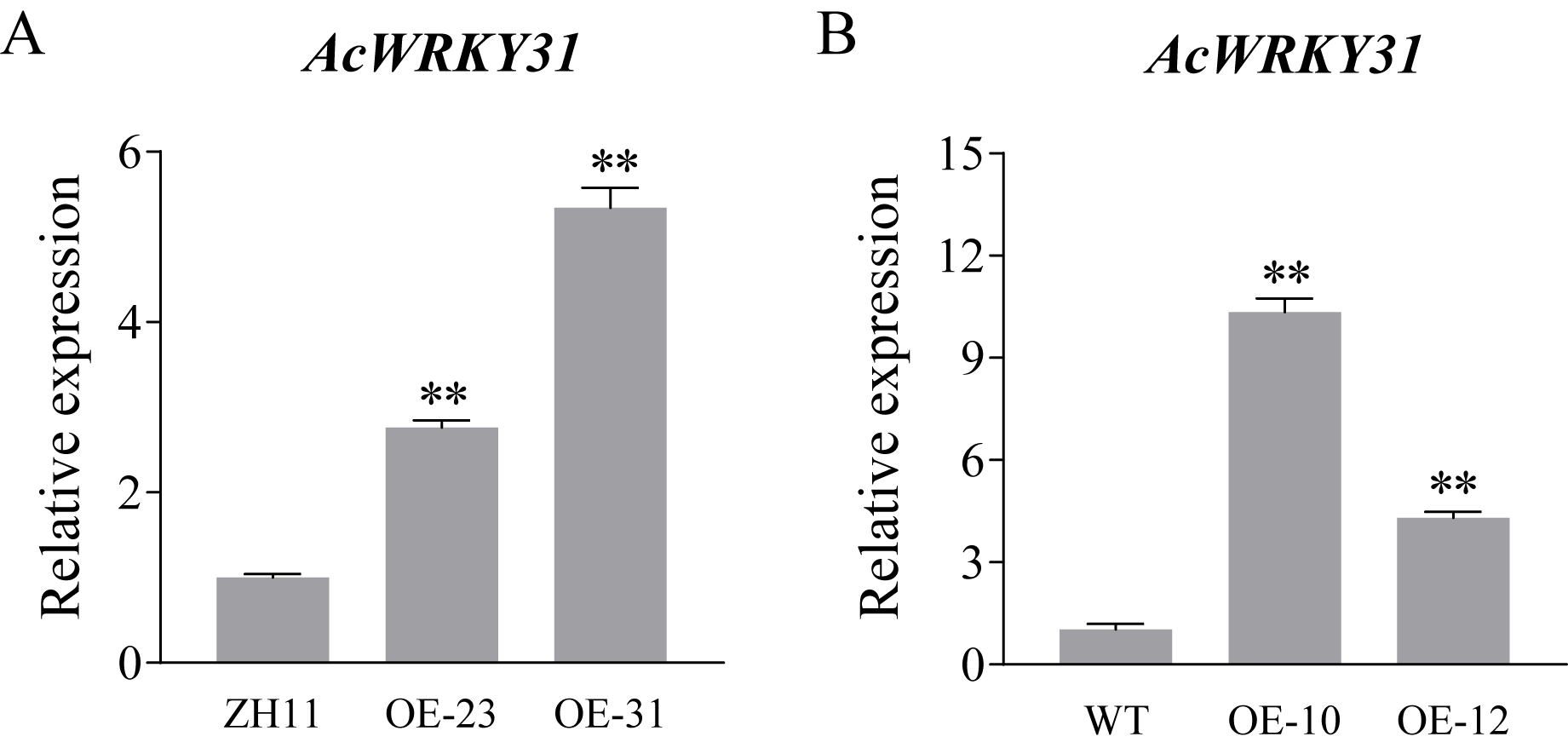

Supplement: Supplementary file 1 [file ijms-23-06269-s001.zip › Supplementary Figure S1.tif]

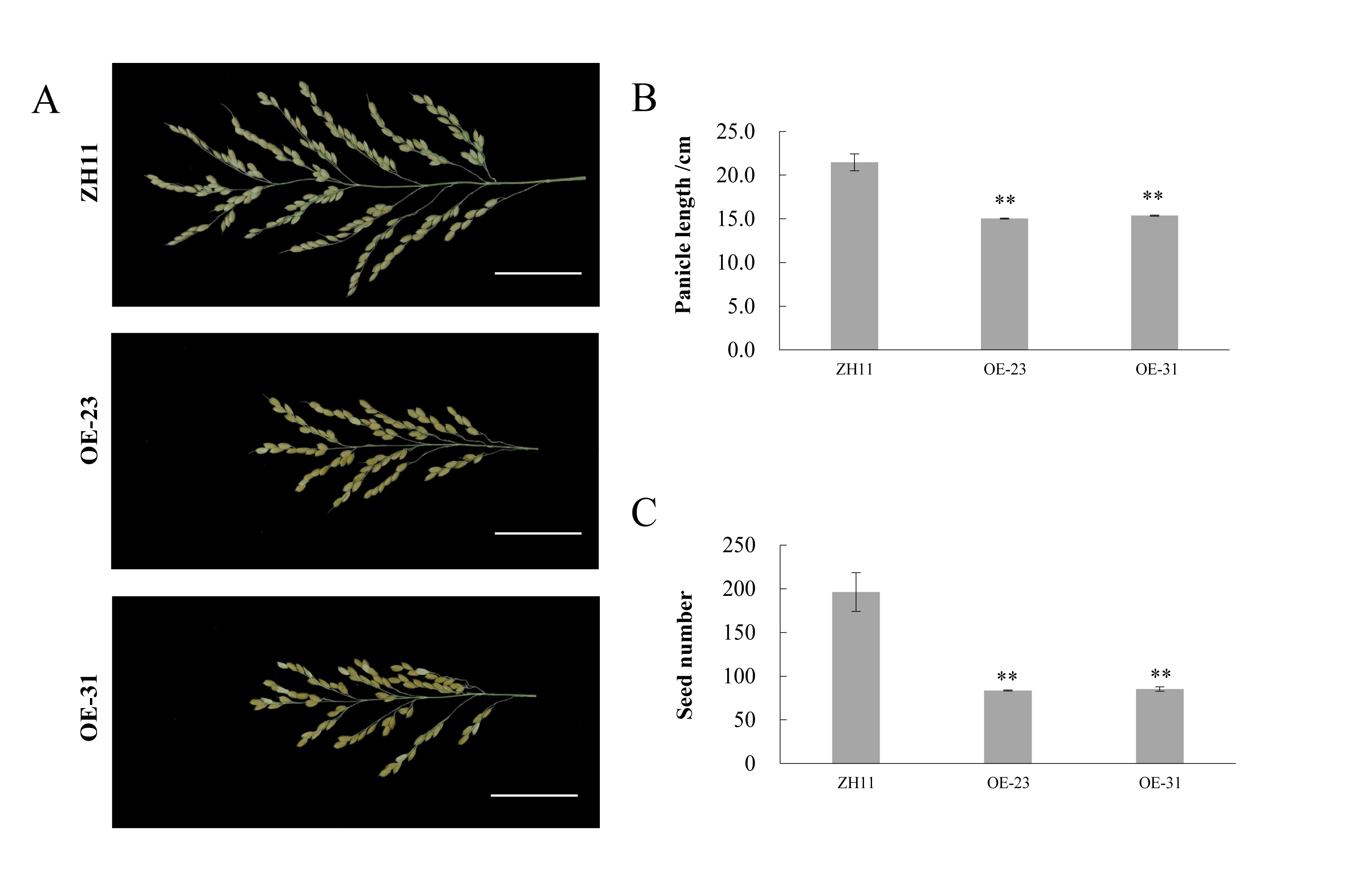

Supplement: Supplementary file 1 [file ijms-23-06269-s001.zip › Supplementary Figure S2.tif]

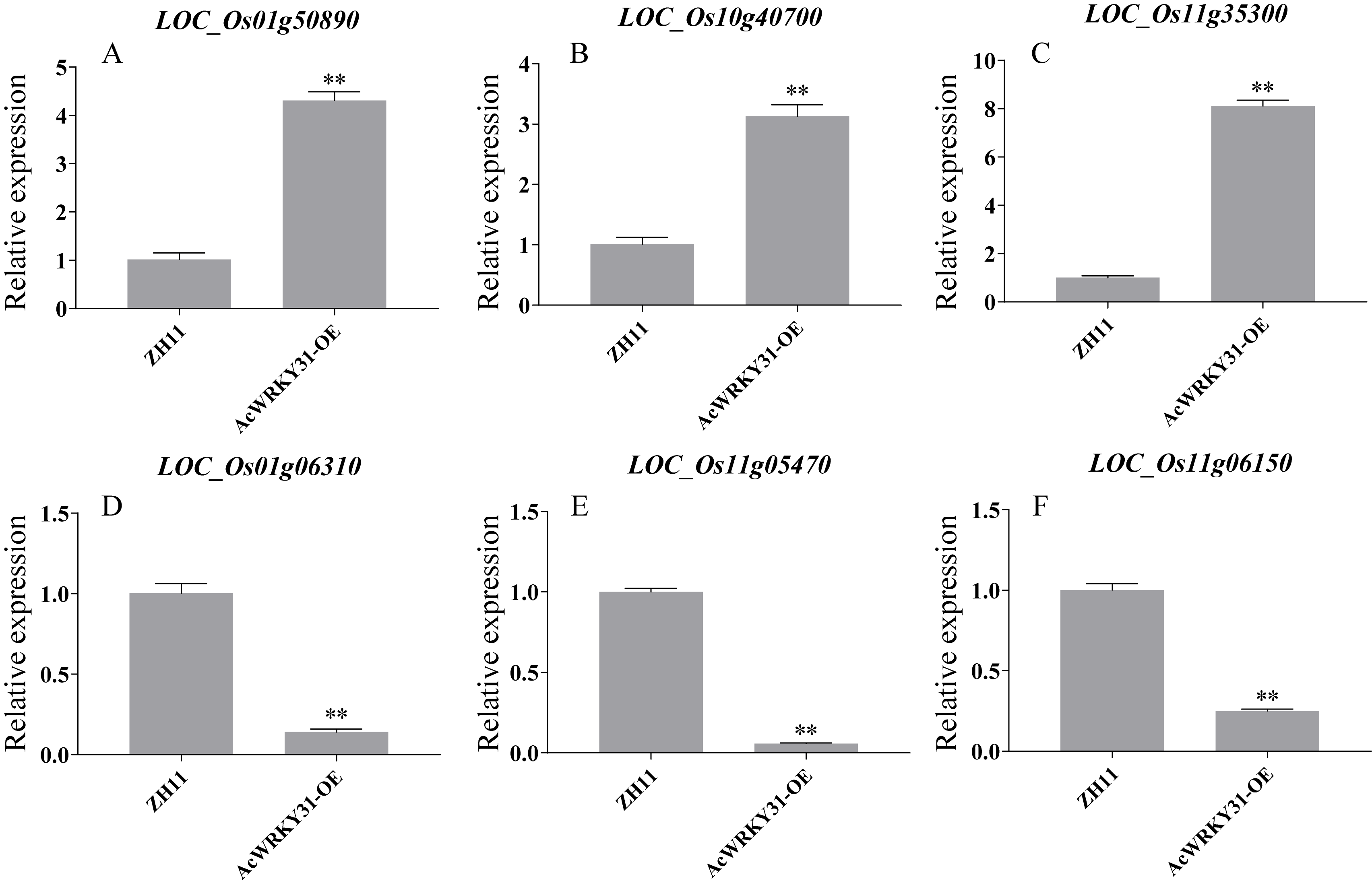

Supplement: Supplementary file 1 [file ijms-23-06269-s001.zip › Supplementary Figure S3.tif]
